# Acknowledgment to the Reviewers of *Sports* in 2022

**DOI:** 10.3390/sports11010018

**Published:** 2023-01-13

**Authors:** Sports Editorial Office

**Affiliations:** MDPI AG, St. Alban-Anlage 66, 4052 Basel, Switzerland

High-quality academic publishing is built on rigorous peer review. *Sports* was able to uphold its high standards for published papers due to the outstanding efforts of our reviewers. Thanks to the efforts of our reviewers in 2022, the median time to first decision was 24 days and the median time to publication was 48 days. Regardless of whether the articles they examined were ultimately published, the editors would like to express their appreciation and thank the following reviewers for the time and dedication that they have shown *Sports*:
Adams, KentKrzysztofik, MichałAhmad, IrshadKubina, MilanAidar, Felipe J.Kubo, MasayoshiAkca, FiratKujawski, SławomirAkyıldız, ZekiLa Torre, AntonioAlberti, GiampietroLane, Michael TimothyAlmeida-Neto, Paulo Francisco DeLanferdini, Fábio JunerAlonso-Fernandez, DiegoLastella, MicheleAmbrozy, TadeuszLathlean, Timothy J. H.Ammendolia, AntonioLatino, FrancescaAndronikos, GeorgeLavender, AndrewAngosto Sánchez, SalvadorLee, Hyun-WooAntúnez, AntonioLeiva-Cepas, FernandoAparicio-Martinez, PilarLemoyne, JeanAparisi, DavidLeslie, Stephen W.Araujo, Rodrigo CappatoLeuciuc, FlorinArboix-Alió, JordiLin, Hui-TingArdigò, Luca PaoloLipert, AnnaArede, JorgeLiu, JingxiaArli, Senay KaradagLochbaum, MarcArslan, ErsanLoh, WeipingAttia, AhmedLopes, HélderAune, Tore KristianLopes, MarioAzevedo, Rui Miguel Simões DeLopes, SílviaAziz, Abdul RashidLópez Bedoya, JesúsBabic, VesnaLópez-de-Celis, CarlosBadau, AdelaLópez-García, SergioBadau, DanaLópez-Laval, IsaacBahenský, PetrLópez-López, DanielBailey, Joshua P.Lorenzetti, SilvioBalci, Sukru SerdarLozano, DemetrioBallmann, ChristopherLuk, Hui-YingBaranauskas, MariusLum, DannyBatatinha, Helena Angélica PereiraMalliaropoylos, NikosBatrakoulis, AlexiosMalsagova, KristinaBehm, DavidMandroukas, AthanasiosBelcic, IvanManzano-Sánchez, DavidBellar, DavidMarcovitch, StuartBerecki‐Gisolf, JannekeMarinaro, CinziaBerger, NicolasMarjoribanks, TimBerki, TamásMarocolo, MoacirBezerra, PedroMarotta, NicolaBielec, GrzegorzMarqués-Jiménez, DiegoBild, WaltherMarqueta, Pedro ManonellesBillat, VeroniqueMartín Valero, RocíoBobo-Arce, MartaMartínez-Jiménez, Eva MaríaBoccia, GennaroMassart, AlainBogdanis, GregoryMassuça, Luís MiguelBoguszewski, DariuszMastalerz, AndrzejBollinger, LanceMatos, SérgioBonanni, RobertoMcLean, BlakeBoolani, AliMcMahon, GerardBostancı, ÖzgürMecías-Calvo, MarcosBottary, RyanMedved, VladimirBougault, ValérieMendo, AntónioBraakhuis, AndreaMendoza-Muñoz, MaríaBrazier, JohnMennitti, CristinaBronikowska, MałgorzataMessias, Leonardo Henrique DalchecoBrustio, Paolo RiccardoMettler, SamuelBruyndonckx, LucMichel, Marina EvrardBunn, JenniferMiskulin, IvanBusquets, AlbertMitu, FlorinBustad, Jacob J.Moesch, KarinCabri, JanMoir, Gavin L.Calleja-Gonzalez, JulioMonteiro, António MiguelCampa, FrancescoMonteiro, CristinaCampolettano, Eamon T.Monteiro, LuísCampo-Prieto, PabloMornieux, GuillaumeCampos-Jara, ChristianMortatti, Arnaldo LuisCarson, FraserMoscatelli, FiorenzoCaruso, LorenzoMotevalli, MohamadCastañeda Babarro, ArkaitzMühlbauer, ThomasCastelli, LuciaNeiva, Henrique P.Castello, JuliaNirengi, ShinsukeCastillo, DanielNounagnon Frutueux, AgbanglaChaves, VictorNowicki, GrzegorzChen, Shing-JyeNuhmani, ShibiliChilibeck, PhilOchi, EisukeChlíbková, DanielaOlmedilla, AurelioChtara, MoktarOrme, PatrickCicchella, AntonioOtsudo, TakahiroCimadoro, GiuseppeOtsuka, MitsuoClaudino, João Gustavo OliveiraOuergui, IbrahimClemente, Filipe ManuelOxford, SamuelCohn, Randy M.Pääsuke, MatiCoimbra, Danilo ReisPalucci-Vieira, LuizColl, Jesús SiquierPanoutsakopoulos, VassiliosCollins, ChristopherParizotto, Nivaldo AntonioCollins, DavidParpa, KoullaCondello, GiancarloPasini, AlbaConstantini, KerenPatikas, DimitriosConti, ValeriaPatsiaouras, AsteriosContreras-Briceño, FelipePavle, JovanovCook, MatthewPavlova, IuliiaCoratella, GiuseppePekas, ElizabethCordellat Marzal, AnaPellegrino, Joseph K.Cortés-Pérez, IrenePenichet-Tomás, AlfonsoCosta, JúlioPethick, JamieCosta, Mário J.Petranović, Matea ZajcCraig, Bruce W.Petrigna, LucaCrespo, MiguelPeyré-Tartaruga, Leonardo AlexandreCrowley-McHattan, ZacharyPhelan, Elizabeth A.Cuadrado-Peñafiel, VíctorPicerno, PietroĆuk, IvanPiepiora, PawełCumming, Sean P.Pimentel, GustavoCynarski, Wojciech J.Pitacho, LilianaCzamara, AndrzejPitchford, NathanDa Costa, TeresaPla, RobinDa Silva Batista, Marco AlexandrePojskic, HarisDadelo, StanislavasPolechoński, JacekDaka, BledarPopovic, StevoDalbo, VincentPraça, GibsonDaliry, AnissaQuesada, Jose Ignacio PriegoDavies, Timothy B.Raabe, JohannesDawe, Robert J.Rahmati, MasoudDe Almeida-Neto, Paulo FranciscoRaiola, GaetanoDe Lima, Leonardo C. RabelloRama, LuísDe Ruiter, Cornelis JohannesRanavolo, AlbertoDemarie, SabrinaRanieri, MaurizioDi Corrado, DonatellaRauter, SamoDiez-Vega, IgnacioRefoyo, IgnacioDomingos, ChristopheReis, JoanaDonahue, PaulReverdito, Riller SilvaDonti, OlyviaRhim, Hye ChangDoorley, James D.Riaza, Alfonso De La RubiaDrid, PatrikRiso, Eva-MariaDuca, MarcoRodriguez, Augusto X.Duglio, StefanoRodríguez-Rosell, DavidĐurković, TomislavRogers, Bruce JeffreyEgan, BrendanRogers, ChristineEken, OzgurRogowska, AleksandraEL Ashker, SaidRomann, MichaelEslami, MansourRomero-Arenas, SalvadorEspada, MárioRomero-Franco, NataliaEvans, Elizabeth S.Rubio-Arias, Jacobo Á.Falup-Pecurariu, OanaRydzik, ŁukaszFarrell, Stephen W.Saavedra, MiguelFattorini, LuigiSadeghi, HassanFernández Gavira, JesúsSaemi, EsmaeelFernández Lázaro, DiegoSáenz-Lopez, PedroFernández-García, José CarlosSalahzadeh, ZahraFessl, IsabellaSánchez Infante, JorgeFilmalter, Sara E.Sánchez-Sánchez, JavierFischetti, FrancescoSantamaría, RafaelFloria, PabloSantos, Fernando Jorge Lourenço DosFominiene, Vilija BiteSanz, Adrián VarelaForte, PedroSarcevic, PeterFoskett, AndrewScanlan, AaronFoti, CalogeroScelles, NicolasFouasson-Chailloux, AlbanSchiltenwolf, MarcusFuller, ScottScudiero, OlgaGabrys, TomaszSelles-Perez, SergioGajewski, JanSelmi, OkbaGallardo, Ana MaríaSempsrott, Justin R.Gamonales, José M.Sharma-Ghimire, PragyaGarcía-Ceberino, Juan ManuelShulman, Lee P.García-Gómez, SalekySiesmaa, EmmaGarcía-Muro San José, FranciscoŠimenko, JožefGarrido, NunoSirico, FeliceGellner, Ryan A.Sklempe Kokic, IvaGentile, AmbraŠkrobot, DubravkaGhigiarelli, Jamie J.Smith, Johneric W.Giannaki, ChristoforosSmith, MatthewGillen, Zachary M.Sobolewski, EricGinevičienė, ValentinaSolana-Tramunt, MonicaGinszt, MichałSousa Neto, Ivo Vieira DeGiovanni, FiorilliSousa, AntónioGirardi, MicheleSousa, FilipaGlebova, EkaterinaSousa-Sá, EduardaGmada, NabilSoylu, YusufGomes Da Rosa, RodrigoSpieszny, MichałGomes, BeatrizStaderini, Enrico MariaGomez-Paniagua, SantiagoStafie, Celina-SilviaGöpfert, BeatStanula, ArkadiuszGrabara, MałgorzataStaunton, Craig A.Grashow, RachelStebbings, GeorginaGraybeal, AustinStojanovic, MarkoGreco, GianpieroStruzik, ArturGrivas, GerasimosStupar, DusanGronek, PiotrSuchomel, TimGu, YaodongSumi, DaichiGualdi-Russo, EmanuelaSvensson, ReneGüllich, ArneSzabo, Dan-AlexandruHackett, DanielTaheri, MortezaHadamus, AnnaTaiar, RedhaHanssen, BrittaTakabayashi, TomoyaHayes, PhilTan, Frankie H. Y.Heinrich, KatieTanaka, TakahiroHenriques-Neto, DuarteTancredi, VirginiaHertting, KristerTcheslavski, Gleb V.Hoffmann, UweThompson, BrennanHokanson, JamesTill, KevinHolliday, WendyTomczak, AndrzejHolmes, CliftonTordi, Nicolas R.Honceriu, CezarToubekis, ArgyrisIlieș, AlexandruTsalis, George A.Inaba, HiromiTschan, HaraldMarinellaIoannou, LeonidasTsoukos, AthanasiosIshida, AiTuesta, MarceloIshido, MinenoriTunér, JanIshikura, KeisukeUehara, YoshinariIto, TadashiUngureanu, Alexandru NicolaeJaszczur-Nowicki, JarosławUthoff, AaronJee, YongseokVaccarezza, MauroJelicic, MarioValdivia-Moral, PedroJennings, GeorgeValero, Alberto FerrizJepkorir Rose, Chepyator-ThomsonVargas Vitoria, RodrigoJoachim, MikelVarillas Delgado, DavidJóhannsdóttir, Kamilla RúnVásquez-Gómez, JaimeJomes, EleriVecchia, Laura Adelaide DallaJukić, IgorVersic, SimeKaczka, PiotrVillafaina, SantosKamiş, OkanVillalba, Marina M.Kang, NyeonjuVink, MarkKaplan, AlanVisser, BartKara, H. VolkanWard, LeighKarninčić, HrvojeWąsik, JacekKhacharem, AïmenWatson, JackKilit, BülentWelt, TobiasKim, Hyun-DuckWhite, Jason B.Kim, KijinWillems, Mark Elisabeth TheodorusKim, KyungyeolWilliams, Morgan DavidKolen, Angela M.Wollmer, Per E.Kolmeder, CarolinWolny, TomaszKonarski, JanWong, Duo Wai-ChiKoozehchian, MajidWu, XiuyunKoropanovski, NenadYildiz, MehmetKostrzewa, MaciejYoshihara, ToshinoriKreider, Richard B.Zaina, FabioKristen, Karl HeinzZaras, NikolaosKról, PiotrZemková, ErikaKrommidas, CharalamposZenic, NatasaKruszewski, Artur


